# Laparoscopic total extraperitoneal (TEP) inguinal hernia repair with preperitoneal closed-suction drainage reduced postoperative complications

**DOI:** 10.1186/s12893-022-01900-9

**Published:** 2023-01-17

**Authors:** Guangbo Wu, Danli Shi, Min Chen, Chihao Zhang, Hongjie Li, Meng Luo, Qiang Fan

**Affiliations:** Department of General Surgery, Shanghai Ninth People’s Hospital, Shanghai Jiao Tong University School of Medicine, Shanghai, China

**Keywords:** Inguinal hernia repair, Drainage, Complications

## Abstract

**Background:**

Although laparoscopic total extraperitoneal (TEP) inguinal hernia repair has the advantages of less bleeding, less trauma, less pain, and fast recovery, there are several issues that need to be addressed. This study aims to evaluate the effectiveness of preperitoneal closed‑suction drainage on reducing postoperative complications in TEP inguinal hernia repair.

**Methods:**

A retrospective analysis of 122 patients who underwent TEP inguinal hernia repair between June 2018 and June 2021 was performed. The patients were divided into the drainage group and the non-drainage group according to whether the drainage tube was placed or not. Clinical data, surgical procedures and outcome of these patients were collected and analyzed to assess the effectiveness of drainage.

**Results:**

A total of 122 patients undergoing TEP surgery were screened, of which 22 were excluded. Most of the patients were male with right indirect inguinal hernia. There was no difference in the mean length of hospital stay between the two groups. Postoperative pain was alleviated by preperitoneal closed‑suction drainage 24 h after operation (*p* = 0.03). The rate of complications such as scrotal edema, seroma and urinary retention in the drainage group was significantly lower than that in the non-drainage group (*p* < 0.05). Multivariate regression analysis showed that drainage was beneficial to reduce postoperative complications (OR, 0.015; 95% CI, 0.002–0.140; p < 0.01). In addition, it was worth noting that in subgroup analysis, patients with hernia sac volume > 10 cm^3^ might receive more clinical benefits by placing drainage tube.

**Conclusion:**

In TEP inguinal hernia repair, placing drainage tube is a simple and feasible traditional surgical treatment, which can promote postoperative recovery without increasing the risk of infection, especially in patients with large hernia sac volume.

**Supplementary Information:**

The online version contains supplementary material available at 10.1186/s12893-022-01900-9.

## Introduction

Inguinal hernia is a common surgical condition, affecting approximately 220 million people worldwide [1]. More than 20 million patients undergo inguinal hernia surgery each year [2]. Laparoscopic inguinal hernia repair is a minimally invasive surgery, such as laparoscopic total extraperitoneal (TEP) inguinal hernia repair, which has become one of the gold standards for inguinal hernia repair [3–5]. However, although TEP surgery has the advantages of less bleeding, less trauma, less pain, and fast recovery, there are several issues that need to be addressed.

Numerous previous studies have reported the complications such as hematoma or seroma, scrotal edema, groin pain and urinary retention after TEP inguinal hernia repair [2, 6–10]. Up to now, despite the better laparoscopic system, more surgical training, and more accurate knowledge of preperitoneal anatomy, surgeons still find that postoperative complications can’t be completely avoided. For example, seroma is a common sequela in the early postoperative period [11], and its prevalence in the first week after surgery is as high as 37.9% [12]. Fortunately, most of the patients with seroma can resolved spontaneously without intervention or only need conservative treatment without surgical intervention. However, it is worth noting that the formation of seroma may lead to the aggravation of postoperative pain, and increase the risk of scrotal edema and urinary retention, which in turn prolong hospital stay.

In addition, many efforts have been made by surgeons to reduce postoperative complications, but there are still some disputes in these methods [13–15]. In 1980, Beacon et al. first reported the method of placing drainage after inguinal hernia repair in a randomized controlled trial. Notably, his results reported that placing drainage was an effective method to reduce surgery-related complications, especially in complex open inguinal hernia repair [16]. Nevertheless, some researchers dispute the use of drainage tubes in previous clinical studies, and even some studies have completely different conclusions. Rodriguez et al. reported that the use of suction drainage in inguinal hernia repair did not provide any benefit [17]. Therefore, in the present study, we aim to investigate the effectiveness of preperitoneal closed‑suction drainage on reducing postoperative complications in TEP inguinal hernia repair.

## Methods

### Patients

A retrospective study was performed from June 2018 to June 2021 in the General Surgery Department of Shanghai Ninth People’s Hospital. A total of 122 patients undergoing TEP inguinal hernia repair with complete reduction of hernia sac were included in this study. All relevant data were collected by the surgeon from the medical record system. All patients were followed up for 3 months after operation. The participants were categorized based on a review of their surgical history and divided into two groups according to whether a drainage tube was placed after an operation.

### Inclusion and exclusion criteria

Inclusion criteria: Patients were age older than 18 years, with a unilateral inguinal hernia, no additional surgical procedures at the time of the inguinal hernia repair, no surgical procedures in preceding 30 days; no other analgesic medication was administered during the postoperative period.

Exclusion criteria: Patients were excluded if they had bilateral or recurrent inguinal hernia, irreducible hernia, incarcerated hernia, or severe co-morbidities.

### Surgical treatment

**Fig. 1:**
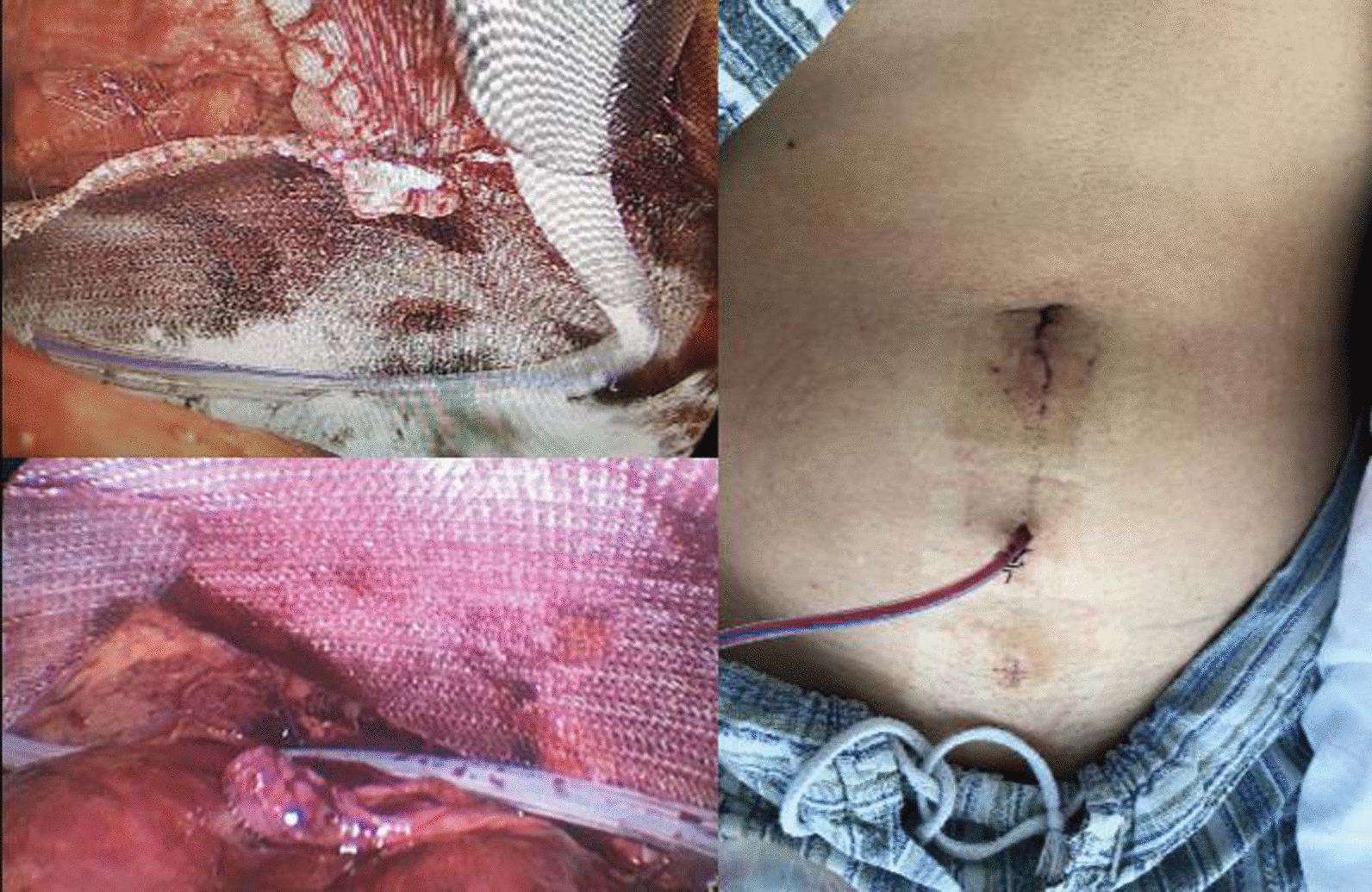
Closed‑suction drainage tubes were placed in the preperitoneal space

TEP inguinal hernia repair was performed in a standard surgical procedure as previously described [18]. A prosthetic mesh was placed in the preperitoneal space through 10 mm intraoperative trocar hole. Bipolar electrocautery was used to control bleeding during the operation. The prosthetic mesh was placed without wrinkles, and covered defects in the Hasselbach region, the indirect ring, the obturator ring and the femoral triangle. Next, in the drainage group, a standard closed‑suction drainage tube was placed in preperitoneal space through the 5 mm intraoperative trocar hole, which was used to place laparoscopic instruments during the operation. Thus, the extra surgical trauma to patients is avoided by using the intraoperative trocar holes. Finally, the surgeon was careful not to displace the mesh, and then deflated and closed the preperitoneal space. On the other hand, in the control group, the drainage tube was not placed, and other procedures were similar. The surgical procedures are shown in Fig. 1.

### Calculation of hernial sac volume

All patients underwent preoperative abdominal computerized tomography (ACT, Philips Brilliance CT Lightspeed-64 multi-slice spiral scanner). According to previous studies, hernia sac volume (HSV) can be approximately regarded as an ellipsoidal structure [19]. In order to estimate the hernia sac volume, anterior–posterior, longitudinal and transverse dimensions of the hernia sac were measured by ACT (A, B and C, respectively, Additional file 1: Fig. S1). Based on these values, the volume formula of ellipsoid is used to calculate HSV: Volume = 4/3 × π × radius A × radius B × radius C. The formula of HSV can be further simplified as: HSV ≈ 0.52 × A × B × C [20, 21]. In addition, during the operation, the surgeons further confirmed that the ACT measurement was consistent with the actual size of HSV.

### Main outcomes and measurements

All demographic and clinical data were collected from our hospital’s medical records, including surgical data (inguinal hernia type, hernia sac volume, operation time, etc.), postoperative drainage fluid and postoperative complications (seroma formation, scrotal edema, inguinal pain, urinary retention and incision infection).

The gravimetric method is a simple, accurate, and clinically practical measurement, which is used to evaluate the blood loss in TEP surgery [22]. According to this method, the weight of the surgical gauze should be weighed before and after the operation. Previous study reported that blood density could be estimated as one gram per milliliter [23]. The difference of gauzes’ weight was considered as an accurate assessment of bleeding volume.

The visual analog scale (VAS) for pain was used to evaluate the perception of pain, on a scale of 0 to 10 with 0 indicating “no pain” and 10 meaning “worst pain”. VAS was performed in both groups at 6 h and 24 h after the operation. Pain score is: 0 = no pain, 1–3 = mild pain, 4–6 = moderate pain, and 7–10 = severe pain.

### Statistical analysis

SPSS 22.0 software (IBM, Armonk, NY, USA) was used for statistical analyses. Quantitative variables were presented as mean ± Standard deviation (SD). Student’s t-test and nonparametric test were used for statistical analysis. Categorical variables including absolute and relative frequencies were tested by Pearson’s Chi-squared or Fisher’s exact. Univariate and multivariate analysis was performed using binary logistic regression. The multivariable regression models were constructed by stepwise addition of covariates shown via simple regression to be significantly associated with the outcome. The level of significance was set to α = 0.05 with 95% confidence interval (CI).

## Results

**Fig. 2:**
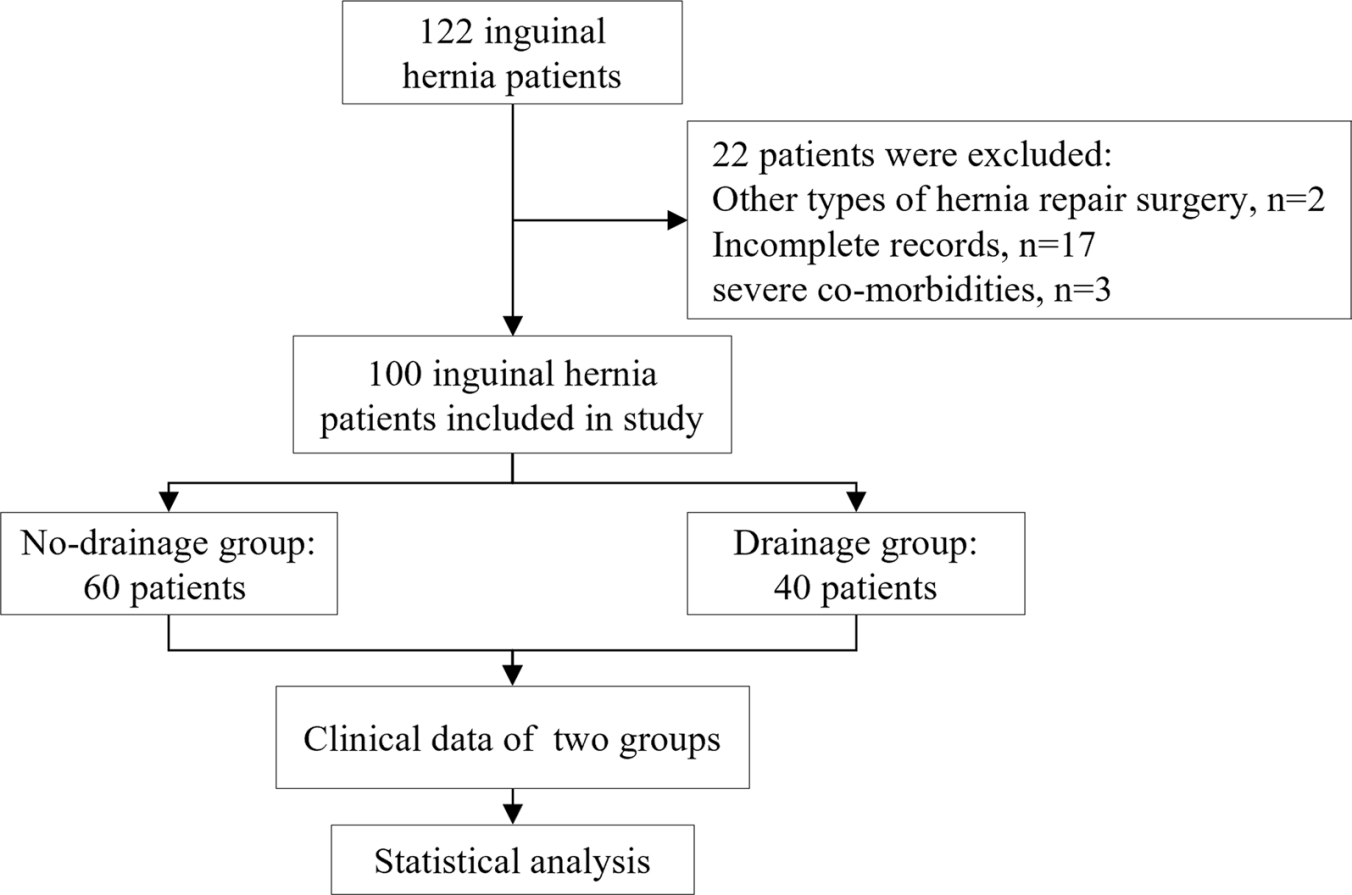
Consort diagram for the study

A total of 122 patients undergoing TEP inguinal hernia repair with complete reduction of hernia sac were screen in this study. Twenty-two patients with other types of hernia repair surgery, incomplete medical records or severe co-morbidities were excluded, as shown in the consort diagram below (Fig. 2). According to whether a drainage tube was placed or not, all patients were divided into the drainage group and the non-drainage group. There were 40 cases in the drainage group. Most of the patients included were male, suffering from right indirect inguinal hernia. There was no difference in the mean length of hospital stay between the two groups (Table 1). Most patients (95%) were discharged from hospital within 3 days after the operation. Only two patients in the drainage group had the longest length of hospital stay at 4 days.

**Table 1:** Patient demographics

Characteristics	Drainage (n = 40)	Non-drainage (n = 60)	t/χ^2^	*p*
Gender, n [%]				
Male	39 [97.5]	57 [95.0]	0.01	0.92
Female	1 [2.5]	3 [5.0]		
Age, years/SD	60.7 ± 15.4	63.3 ± 13.9	0.85	0.40
Type of hernia, n [%]				
Indirect inguinal hernia	33 [82.5]	53 [88.3]	0.68	0.41
Direct inguinal hernia	7 [17.5]	7 [11.7]		
Side of hernia, n [%]				
Right side	28 [70.0]	42 [70.0]	< 0.01	1.00
Left side	12 [30.0]	18 [30.0]		
Inpatient stay, days/SD	2.1 ± 0.9	2.0 ± 0.8	0.48	0.63

The TEP inguinal hernia repair was performed as described above. The hernia sac volume in the drainage group tended to be larger than that in the non-drainage group. However, we did not observe the differences of mean operative time or blood loss between the two groups. As shown in Table 2, the mean drainage volume of drainage group was 103.6 ± 66.0 ml. The drainage volume was usually markedly reduced at 48 h after TEP inguinal hernia repair. Therefore, the drainage tube was removed on the second day after the operation.

**Table 2:** Operative details

Characteristics	Drainage (n = 40)	Non-drainage (n = 60)	t/Z	*p*
Hernia sac volume, cm^3^	8.5 (4.5–20.0)	6.0 (4.0–12)	− 1.41	0.16
Operation time, min/SD	43.3 ± 15.2	40.8 ± 13.5	0.83	0.41
Blood loss, ml/SD	8.6 ± 4.5	9.1 ± 4.1	− 0.52	0.60
Drainage volume, ml/SD	103.6 ± 66.0	–	–	–

**Fig. 3:**
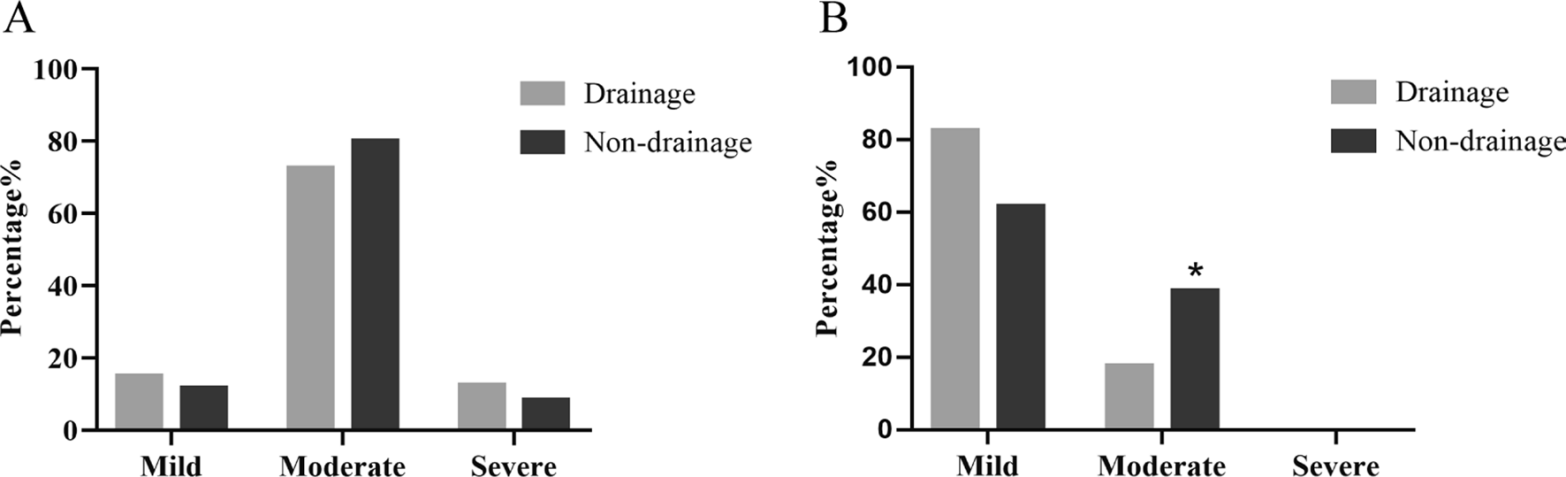
Percentages of VAS pain score after operation. **A** The VAS pain score at 6 h after operation. **B** The VAS pain score 24 h after operation (*p < 0.05)

The VAS scores were collected at 6 h and 24 h after operation in both groups. At 6 h postoperatively, most of the patients reported moderate pain. The percentages of mild and severe pain in the drainage group were 15% and 12.5%, respectively, while those in the non-drainage group were 11.7% and 8.3%, respectively (Fig. 3A). Moreover, 24 h after operation, the pain of most patients was relieved, and the proportion of mild pain remarkably increased, with 82.5% in the drainage group and 61.7% in the non-drainage group. Notably, the moderate pain in the drainage group was significantly less than that in the non-drainage group (Fig. 3B). Therefore, there was a difference in VAS pain score between the two groups 24 h after operation (Table 3).

**Table 3:** Pain score

Pain	Drainage (n = 40)	Non-drainage (n = 60)	χ^2^	*p*
6 h postoperative				
No (0)	0	0		
Mild (1–3)	6 [15.0]	7 [11.7]		
Moderate (4–6)	29 [72.5]	48 [80.0]	0.80	0.67
Severe (7–10)	5 [12.5]	5 [8.3]		
24 h postoperative				
No (0)	0	0		
Mild (1–3)	33 [82.5]	37 [61.7]		
Moderate (4–6)	7 [17.5]	23 [38.3]	5.0	0.03*
Severe (7–10)	0	0		

^*^p < 0.05

The postoperative complications of the two groups are shown in Table 4. The rate of scrotal edema in drainage group was significantly lower than that in non-drainage group. In the drainage group, the formation of seroma was markedly reduced. Meanwhile, the rate of urinary retention in the drainage group was significantly lower than that in non-drainage group. On the other hand, the wound infection rate of the two groups was similar, and there was no mesh infection in both groups.

**Table 4:** Postoperative complications

Characteristics	Drainage (n = 40)	Non-drainage (n = 60)	t/χ^2^	*p*
Postoperative temperature, ℃/SD	37.0 ± 0.5	37.1 ± 0.5	0.73	0.47
Postoperative complications, n [%]				
Scrotal edema	3 [7.5]	14 [23.3]	4.26	0.04*
Seroma formation	2 [5.0]	12 [20.0]	4.49	0.03*
Urinary retention	1 [2.5]	9 [15.0]	4.17	0.04*
Wound infection	1 [2.5]	2 [3.3]	< 0.01	1.00
Mesh infection	0 [0.0]	0 [0.0]	–	–

^*^p < 0.05

**Fig. 4:**
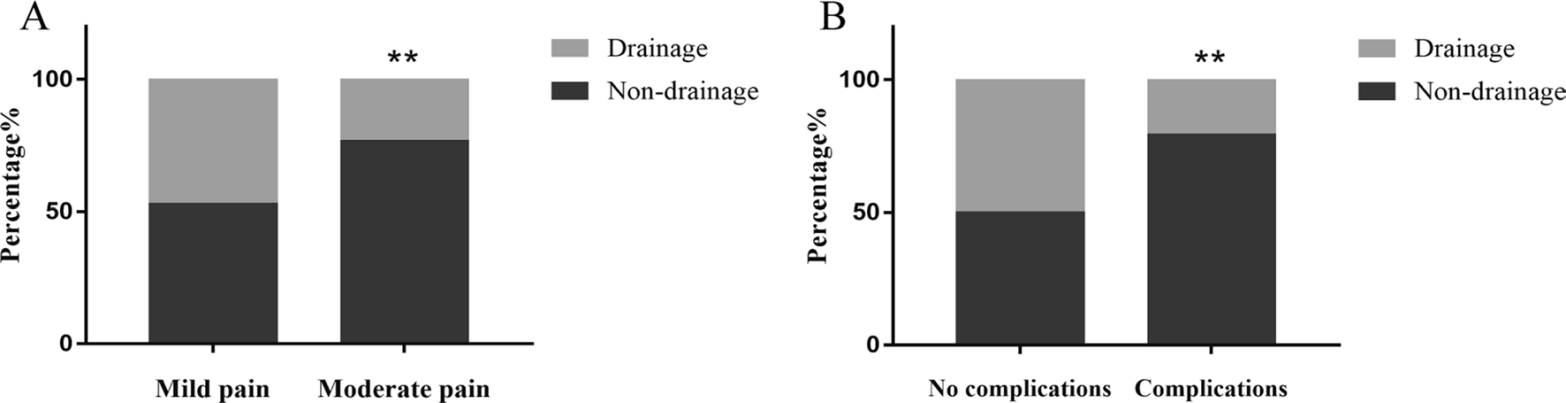
The VAS pain score at 24 h and postoperative complications. **A** The proportion of patients with mild and moderate pain. **B** The proportion of patients with or without complications (**p < 0.01)

In order to further assess the effectiveness of preperitoneal closed‑suction drainage, a regrouping analysis was performed based on the 24-h VAS pain score and complications. There was no difference in the proportion of patients with mild pain between the two groups. However, the proportion of patients with moderate pain in the non-drainage group was higher than that in the drainage group (Fig. 4A). In this study, there were 34 patients with postoperative complications. Only one patient underwent surgical intervention due to the failure of conservative treatment for complications. The proportion of patients with complications in the non-drainage group was higher than that in the drainage group (Fig. 4B). Furthermore, the univariate regression analysis revealed that placement of drainage significantly reduced the risk of complications (OR, 0.259; 95% CI, 0.099–0.678; p < 0.01). The multivariate regression analysis was performed on the potential factors, including drainage placement, hernia sac volume, operation time and blood loss. The results showed that the drainage was beneficial to reduce postoperative complications (OR, 0.015; 95% CI, 0.002–0.140; p < 0.01). In addition, we also found that hernia sac volume was associated with the postoperative complications (OR, 1.418; 95% CI, 1.213–1.657; p < 0.01).

**Fig. 5:**
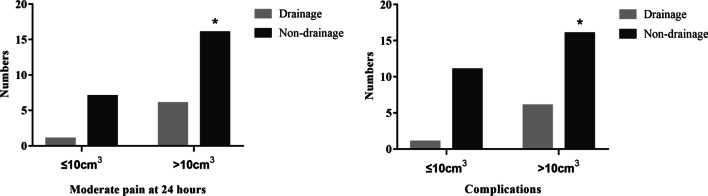
Moderate pain and postoperative complications in subgroup analysis (*p < 0.05)

Considering that hernia sac volume may also be related to complications, a subgroup analysis was used to investigate the relationship between hernia sac volume and clinical benefits of placing drainage. Based on the mean volume of hernia sac, patients were divided into two subgroups: hernia sac volume ≤ 10 cm^3^ and > 10 cm^3^. As shown in Fig. 5, there was a trend for drainage tubes to reduce pain and complications in patients with hernia sac volumes ≤ 10 cm^3^. It was worth noting that patients with hernia sac volume > 10 cm^3^ received more clinical benefits by placing drainage, which could significantly relieve postoperative pain and reduce complications.

## Discussion

Compared with traditional laparotomy, laparoscopic inguinal hernia repair has the advantages of less trauma, faster recovery and lower recurrence rate [2]. TEP inguinal hernia repair is one of the most suitable methods as it is in conformity with physiology and anatomy [24]. In this study, a total of 122 patients undergoing TEP inguinal hernia repair were screened, and 22 patients were excluded. Most of the patients included were male, suffering from right indirect inguinal hernia. Forty cases were in the drainage group. There was no difference in the mean length of hospital stay between the two groups. Postoperative pain was alleviated by preperitoneal closed‑suction drainage 24 h after operation. The rate of complications such as scrotal edema, seroma and urinary retention in the drainage group was significantly lower than that in the non-drainage group. It was worth noting that patients with hernia sac volume > 10 cm^3^ might receive more clinical benefits by placing drainage tubes, which significantly reduced pain and complications. To our knowledge, this is the first study to investigate the effectiveness s of preperitoneal closed‑suction drainage and hernia sac volume on complications in TEP inguinal hernia repair.

The formation of seroma is defined as the exudation and accumulation of fluid in the operation area. In previous studies, numerous interventions have been attempted to minimize its formation, including closure of the dead space with braided sutures, administration of fibrin glue, compression and cauterization of the operation area [25–27]. However, fibrin glue is expensive, and endoscopic suture or cauterization is technically demanding and potentially hazardous to cord structures. It is also inconvenient to use pressurize dressings in that inguinal area. In contrast, drainage is a simpler and cheaper method to reduce the formation of seroma. Our results showed that placing the drainage tube through the 5 mm intraoperative trocar hole did not increase the trauma of patients. Meanwhile, the closed-suction drainage tube can promote the early collapse of the dead space, which leads to early adhesion formation in the preperitoneal space to facilitate fixation of the mesh. Especially for TEP repair studies of unfixed mesh, early adhesion formation can prevent mesh displacement and reduce recurrence [28, 29].

**Fig. 6:**
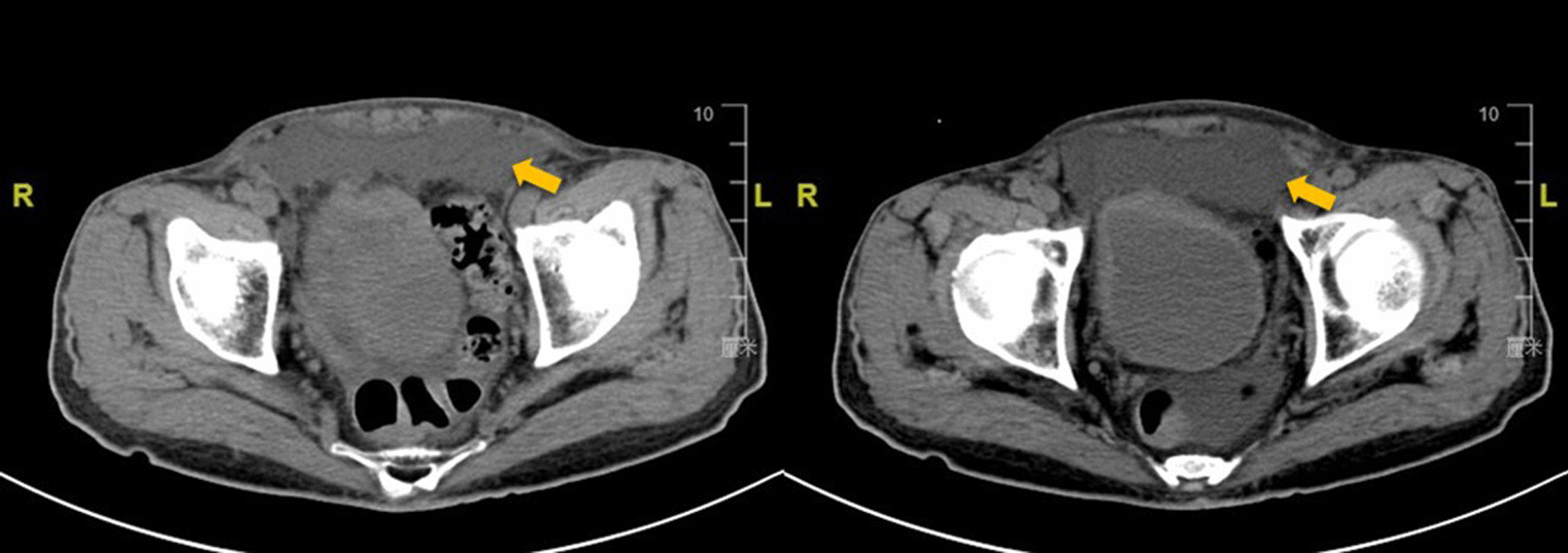
Fluid accumulation in the preperitoneal space

Although most seroma do not cause serious harm to patients, it is still a risk factor leading to other postoperative complications. In our study, the closed-suction drainage tube in the preperitoneal space was used to drain the accumulated fluid, which significantly relieved postoperative pain and reduced scrotal edema, urinary retention and other complications. Our findings are consistent with previous studies [25, 30, 31]. In addition, the postoperative complications also increased psychological burden on patients. In the present study, only one patient experienced severe pain and massive seroma formation after the operation. After conservative treatment failed, the second laparoscopic surgery was performed to drain the fluid in the preperitoneal space (Fig. 6). Therefore, we attempted to solve these clinical problems by placing the closed-suction drainage tube, which is a simple, inexpensive, and traditional method.

In TEP inguinal hernia repair, the risk of infection is the main concern for surgeons, who are reluctant to place a drainage tube in the preperitoneal space. In the guideline on TEP treatment of inguinal hernia by the International Endohernia Society in 2015, there was level 3 evidence that the use of drainage tubes increased the risk of infection or recurrence. Only grade C evidence showed that a closed-suction drainage tube could reduce the risk of seroma formation without increasing the risk of infection [3]. But in 2018, Fan et al. performed a prospective double-blind randomized controlled trial. There was Grade 1 evidence that a closed-suction preperitoneal drain prevented seroma formation without increasing the risk of infection or septic complications [30]. Our study also confirmed that the placement of drainage does not increase the risk of postoperative infection. Standard surgical procedures, closed-suction drainage with negative pressure and postoperative nursing care can effectively reduce the risk of infection.

Postoperative complications, such as seroma, inguinal pain and urinary retention, are associated with surgical trauma and exudation in the operation area. Although large incisions and surgical trauma are avoided by laparoscopic surgery, more severe swellings and exudation in the inguinal region may be caused by larger hernia sac [32, 33]. According to the subgroup analysis of hernia sac volume, we observed that there were significant differences in the clinical benefits of placing drainage in patients with different hernia sac volumes. Patients with hernia sac volume > 10cm^3^ obtained more benefits by placing drainage, which remarkably relieved pain and reduced complications. Based on these findings, our study confirmed that there are greater clinical benefits from placing a drainage tube in patients with larger hernia sac volume. Indeed, the volume of hernia sac may be an important criterion for drainage in patients undergoing TEP inguinal hernia repair.

## Conclusion

In TEP inguinal hernia repair, preperitoneal closed‑suction drainage is associated with the reduction of postoperative pain and complications. The placement of drainage tube is a simple and feasible traditional surgical treatment, which can promote postoperative recovery without increasing the risk of infection, especially in patients with large hernia sac volume.

## Supplementary Information

**Additional file 1: Table S1 **Comparison of postoperative complications. **Fig. S1 **Ellipsoid and the diameters A, B and C.

## Data Availability

The datasets of the current study are available from the corresponding author upon reasonable request.
