# Supplementary material for: Laparoscopic total extraperitoneal (TEP) inguinal hernia repair with preperitoneal closed-suction drainage reduced postoperative complications
Source: BMC Surg. 2023 Jan 17;23:14. doi: 10.1186/s12893-022-01900-9 (PMC9847164; doi:10.1186/s12893-022-01900-9)

**Additional file 1**

**Table S1 Comparison of postoperative complications**

| Complications, n [%] | Drainage (n=40) | | Non-drainage (n=60) | |
| --- | --- | --- | --- | --- |
|  | Yes | No | Yes | No |
| Scrotal edema | 3 [7.5] | 37 [92.5] | 14 [23.3] | 46 [76.7] |
| Seroma formation | 2 [5.0] | 38 [95.0] | 12 [20.0] | 48 [80.0] |
| Urinary retention | 1 [2.5] | 39 [97.5] | 9 [15.0] | 51 [85.0] |
| Wound infection | 1 [2.5] | 39 [97.5] | 2 [3.3] | 58 [96.7] |
| Mesh infection | 0 [0.0] | 40 [100.0] | 0 [0.0] | 60 [100.0] |

**Figure S1 Ellipsoid and the diameters A, B and C.**


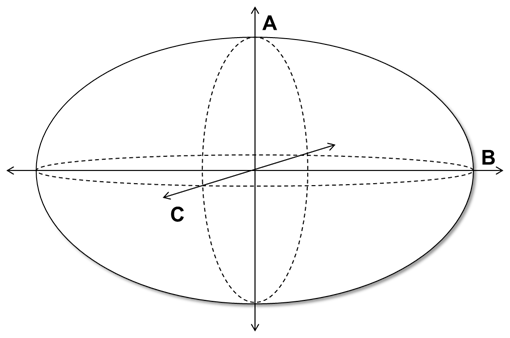

Supplement: Supplementary file 1 — Additional file 1: Table S1 Comparison of postoperative complications. Fig. S1 Ellipsoid and the diameters A, B and C. [file 12893_2022_1900_MOESM1_ESM.docx]
